# Effect of Accelerated Aging on Some Mechanical Properties and Wear of Different Commercial Dental Resin Composites

**DOI:** 10.3390/ma14112769

**Published:** 2021-05-23

**Authors:** Jonne Oja, Lippo Lassila, Pekka K. Vallittu, Sufyan Garoushi

**Affiliations:** 1 Department of Biomaterials Science and Turku Clinical Biomaterial Center—TCBC, Institute of Dentistry, University of Turku, 20500 Turku, Finland; jonne.j.oja@utu.fi (J.O.); liplas@utu.fi (L.L.); pekval@utu.fi (P.K.V.); 2 City of Turku Welfare Division, Oral Health Care, 20101 Turku, Finland

**Keywords:** resin composite, hydrothermal aging, wear, expiration date

## Abstract

The aim of current in vitro research was to determine the effect of hydrothermal accelerated aging on the mechanical properties and wear of different commercial dental resin composites (RCs). In addition, the effect of expiration date of the composite prior its use was also evaluated. Five commercially available RCs were studied: Conventional RCs (Filtek Supreme XTE, G-aenial Posterior, Denfil, and >3y expired Supreme XTE), bulk-fill RC (Filtek Bulk Fill), and short fiber-reinforced RC (everX Posterior). Three-point flexural test was used for determination of ultimate flexural strength (*n* = 8). A vickers indenter was used for testing surface microhardness. A wear test was conducted with 15,000 chewing cycles using a dual-axis chewing simulator. Wear pattern was analyzed by a three-dimensional (3D) noncontact optical profilometer. Degree of C=C bond conversion of monomers was determined by FTIR-spectrometry. The specimens were either dry stored for 48 h (37 °C) or boiled (100 °C) for 16 h before testing. Scanning electron microscopy (SEM) was used to evaluate the microstructure of each material. Data were analyzed using ANOVA (*p* = 0.05). Hydrothermal aging had no significant effects on the surface wear and microhardness of tested RCs (*p* > 0.05). While flexural strength significantly decreased after aging (*p* < 0.05), except for G-aenial Posterior, which showed no differences. The lowest average wear depth was found for Filtek Bulk Fill (29 µm) (*p* < 0.05), while everX Posterior and Denfil showed the highest wear depth values (40, 39 µm) in both conditions. Passing the expiration date for 40 months did not affect the flexural strength and wear of tested RC. SEM demonstrated a significant number of small pits on Denfil’s surface after aging. It was concluded that the effect of accelerated aging may have caused certain weakening of the RC of some brands, whereas no effect was found with one brand of RC. Thus, the accelerated aging appeared to be more dependent on material and tested material property.

## 1. Introduction

In dentistry, resin composites (RCs) have become the most common materials for direct restorations as a result of recent improvements in their physical, bonding, and aesthetic properties [1]. RC consists of reinforcing glass particles or fibers (fillers) embedded in a resin-polymerized matrix. The majority of the composite is made up of these fillers, which vary in size and composition between RCs [1]. These fillers are coated with a silane coupling agent to provide a chemical bond with the resin matrix [1]. Resin matrix are typically based on photosensitive monomers (dimethacrylate), such as bisphenol A-glycidyl methacrylate (Bis-GMA), triethylene glycol dimethacrylate (TEGDMA), and urethane dimethacrylate (UDMA) [1,2]. 

Despite the fact that dental practitioners in public dental care spend more than half of their time applying direct light-cure RCs [3], composite failure rates for high caries risk patients can be as high as 4.6% a year, with estimated lifetimes as short as six years [4,5]. Moreover, the bulk of all restorations are replacements for existing restorations that have failed [6]. In the oral cavity, composite restorations are subjected to a wide range of variable cyclic fatigue loadings under many different aging conditions during service life. Matrix and/or filler degradation, interfacial debonding, microcracking, and/or filler particle fracturing may happen in dental composites as consequence of mechanical and/or environmental aging [7]. Continuous mechanical and environmental aging results in progressive deterioration, crack formation, and catastrophic failure of composite restorations. Many researches are available in literature, focusing on the effect of different aging environments on dental composites [8,9,10]. 

Water aging seems to increase the pull-out of the filler particle on the fractured surface, likely due to weakening of the silane bond between the filler particle and the resin [11]. There are many theories regarding the effect of water aging on dental composite and the impact of water storage and hydration on the performance of dental composites can be significant or insignificant [12]. Depending on ingestion foods and drinks, the temperature extremes in the oral cavity and around restorations have been measured along the range 0.4–77.4 °C [13]. Therefore, in order for the composite restorations to fulfill their tasks effectively, it is of great importance to determine their behaviors under extreme environmental factors. In fact, hydrothermal stability has a critical role in the durability of composite restorations. Although, some authors reported that mechanical properties of particle or fiber-reinforced dental RCs degrade after extreme accelerated hydrothermal aging in boiling water [14,15], further investigation on the newer materials in the market is still required. 

To decide if a RC product has a shelf stability and grant an expiration date, a variety of characteristics should be evaluated. As a result, the substance must be examined to see if it is vulnerable to deterioration that will result in functional failure, as well as the amount of danger that such a failure would pose [16]. Dental practitioners are unaware of the risks of using RCs after their expiration dates. With reference to the above considerations, the aim of this in vitro study was to determine the effect of hydrothermally accelerated aging on the mechanical properties and wear of different commercial dental RCs. In addition, the effect of expiration date of the RC prior its use was also evaluated. The null hypothesis was that after hydrothermal accelerated aging and expiration date, there would be no difference in their tested parameters between the tested resin composites.

## 2. Materials and Methods

A total of five commercial packable RCs were investigated (Table 1). Conventional RCs (Filtek Supreme XTE, G-aenial Posterior, Denfil, and >3 year expired Supreme XTE), bulk-fill RC (Filtek Bulk Fill), and short fiber-reinforced RC (everX Posterior). 

### 2.1. Mechanical Test

Flexural strength (FS) and flexural modulus (FM) for each RC were tested by performing a three-point bending test. Bar-shaped specimens (3 × 4 × 25 mm^3^) were made in a silicone mold with a clear Mylar sheet and a glass slide on top. The RCs were polymerized for 20 s in five different overlapping parts from both sides of the specimen using a hand light-curing device (Elipar S10, 3M ESPE, St. Paul, MN, USA). The light irradiance values (1600 mW/cm^2^) and wavelength spectra (range 430–480 nm) of the light-curing unit were measured using Marc Resin Calibrator (BlueLight Analytics Inc., Halifax, NS, Canada). From each RC, specimens (*n* = 8/group) were either dry stored for 48 h (37 °C) or aged before testing. The specimens were aged in boiling deionized distilled water for 16 h. In order to do this, the specimens were placed in glass bottles of 40 mL distilled water and their caps were tightened. The bottles were then set in a heating oven for 16 h at 100 °C. In line with ISO 4049 (test span: 20 mm, cross-head speed: 1 mm/min, indenter: 2 mm diameter), three-point bending test was assessed. All specimens (*n* = 8/group) were loaded into a universal testing machine (LRX model, Lloyd Instruments Ltd., Fareham, UK) and PC software (Nexygen 4.0, Lloyd Instruments Ltd.) recorded the load-deflection curves. Flexural strength and flexural modulus were measured based on the following equations:

FS = 3F_m_L/(2bh^2^)     FM = SL^3^ /(4bh^3^),

where Fm is the applied load (N) at the highest point of a load-deflection curve, h is the thickness of test specimens, b is the width of test specimens, and S is the stiffness (N/m), where L is the span length (20 mm). S = F/d, where d is the deflection at a point in the straight-line portion of the trace corresponding to load F.

### 2.2. Surface Microhardness 

The surface microhardness of aged (boiling; as described earlier) and not aged (dry) specimens (fractured Bar-shaped) was assessed by a Struers Duramin hardness microscope (Struers, Copenhagen, Denmark) using a load of 1.96 N subjected for 10 s and with 40 objective lenses. Three measurements were taken on the top side of each specimen (*n* = 5 per group). The computer measured the diagonal length impressions and converted Vickers values into hardness values. The following formula was used to determine surface microhardness:

H= (1854.4×P)/d^2,

where *H* is Vickers hardness in kg/mm^2^, *P* is the load in grams and *d* is the diagonals length in μm. 

### 2.3. Double Bond Conversion

The double bond conversion (DC%) was monitored during and after the photoinitiation of polymerization using Fourier transform infrared spectroscopy (FT-IR) (Spectrum One, Perkin-Elmer, Beaconsfield Bucks, UK) with an attenuated total reflectance (ATR) accessory. The RCs were tested in a 1.5 mm thick and 4.5 mm diameter mold. The spectrum of the unpolymerized specimen was first placed and measured in the mold. Using the same curing protocol, the specimen was then irradiated via an upper glass slide. When irradiated, the specimen was scanned for its FT-IR spectrum (monitored for 15 min). The DC% was measured from the aliphatic C=C peak at 1636 cm^−1^ and normalized against the aromatic ring C=C peak at 1608 cm^−1^ based on this equation:

$$DC=\frac{{\left(A_{c=c}/A_{ph}\right)}_{0}-{\left(A_{c=c}/A_{ph}\right)}_{t}}{{\left(A_{c=c}/A_{ph}\right)}_{0}}\times 100\%,$$

where *A_C=C_* and *A_ph_* were the absorbance peak area of methacrylate C=C at 1636 cm^−1^ and aromatic ring at 1608 cm^−1^, respectively; *(A_C=C_/A_ph_)*_0_ and *(A_C=C_/A_ph_)_t_* represented the normalized absorbency of the functional group at the radiation time of 0 and *t*, respectively; *DC* is the conversion of methacrylate *C=C* at a given radiation time. Five trials were carried out for each tested RC.

### 2.4. Two-Body Wear 

Four specimens of each RC were prepared in an acrylic resin block for localized wear test. For each group, long cavity (20 mm length × 10 mm width × 2 mm depth) was prepared in and then RC was applied in a bulk layer into the prepared cavity and covered with a glass slide before being light-cured with same used protocol in five different positions. The surfaces were then polished flat with a series of #1200 to #4000 grit silicon carbide sheets. The specimens from each RC were either dry stored for 48 h (37 °C) or aged (boiling; as described earlier) before testing. The chewing simulator (CS-4.2, SD Mecha-tronik, Feldkirchen-Westerham, Germany) was used for the two-body wear test, which has two chambers that simulate vertical and horizontal motions with water at the same time. Every chamber contains a lower plastic sample holder in which the RC specimen was embedded, as well as an upper sample holder with a screw to secure the loading tip (antagonistic). The manufacturer’s standard loading tips (Steatite ball, 6 mm) were embedded in acrylic resins and then secured with a fastening screw in the upper sample holders. A 2 kg weight was used, which corresponded to 20 N of chewing force and 15,000 loading cycles at 1.5 Hz. On the surface of each specimen, the wear patterns (*n* = 6/group) were scanned with 3D optical microscope (Bruker Nano GmbH, Berlin, Germany) using Vision64 software. Total wear depth values (μm) were measured from different points, reflecting the average of the lowest or deepest points of all profile scans. 

### 2.5. Microscopic Analysis

The effect of aging on the surface of RCs was evaluated by scanning electron microscopy (SEM, JSM 5500, Jeol Ltd., Tokyo, Japan). Two fractured specimens (Bar-shaped) from each RC were randomly selected for the evaluation. Before observation, all the specimens were coated with a gold layer in a vacuum evaporator using a sputter coater (BAL-TEC SCD 050 Sputter Coater, Balzers, Liechtenstein).

### 2.6. Statistical Analysis

The resulted data were statistically analyzed using two-way analysis of variance (ANOVA) followed by Tukey HSD test (α = 0.05) to assess the differences between the groups using SPSS version 23 (SPSS, IBM Corp., Armonk, NY, USA). Aging and material type were independent variables.

## 3. Results

**Figure 1:**
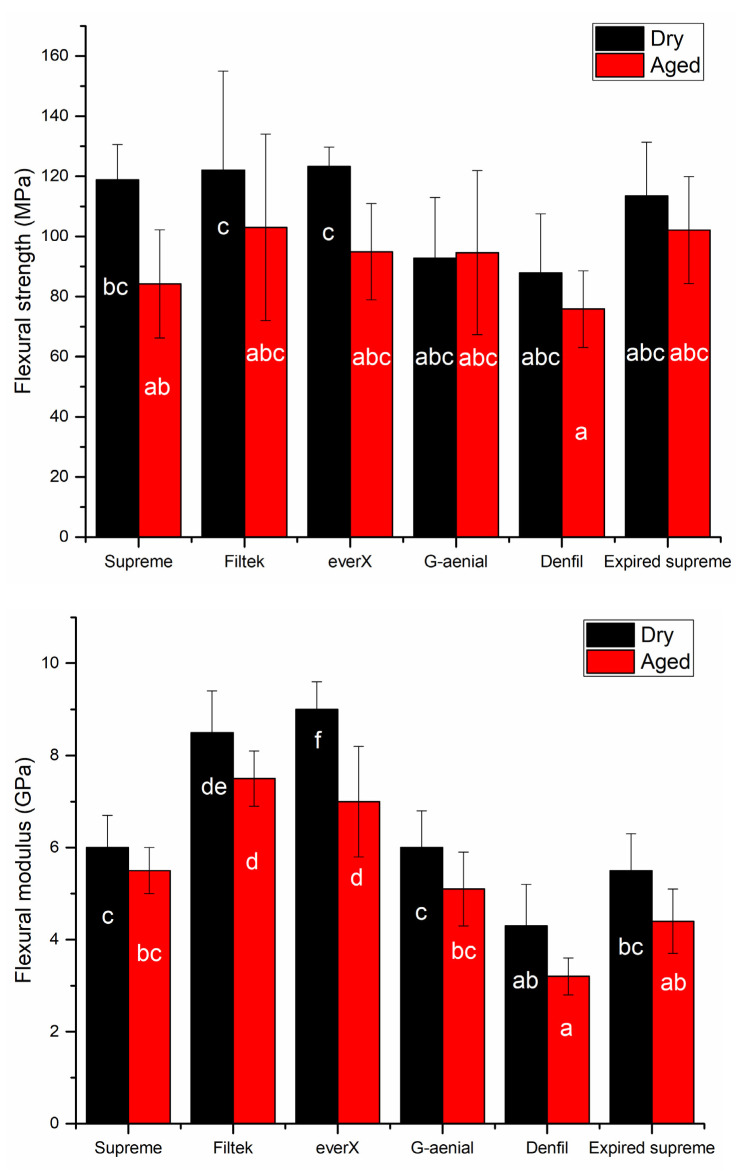
Bar graph showing means flexural strength (MPa) and flexural modulus (GPa) with standard deviations (SD) of tested resin composites. Non-statistically relevant variations (*p* > 0.05) between the materials are represented by the same letters within the bars.

The results of flexural strength (FS) and flexural modulus (FM) of investigated RCs are shown in Figure 1. FS and FM significantly decreased after hydrothermal accelerated aging (*p* < 0.05), except for G-aenial Posterior, which showed no differences in FS. Filtek Bulk Fill and everX Posterior presented the highest FS (122, 123 MPa) in dry condition among all tested RCs. On the other hand, Denfil had the lowest FS values (76 MPa) after aging. According to the results, the expiry date (more than 3 years) has no significant impact on the tested flexural properties (*p* > 0.05). Denfil exhibited the lowest FM (4.3, 3.2 GPa) in both conditions while everX Posterior presented the highest (*p* < 0.05) (9 GPa) in dry condition among the RCs tested. 

**Figure 2:**
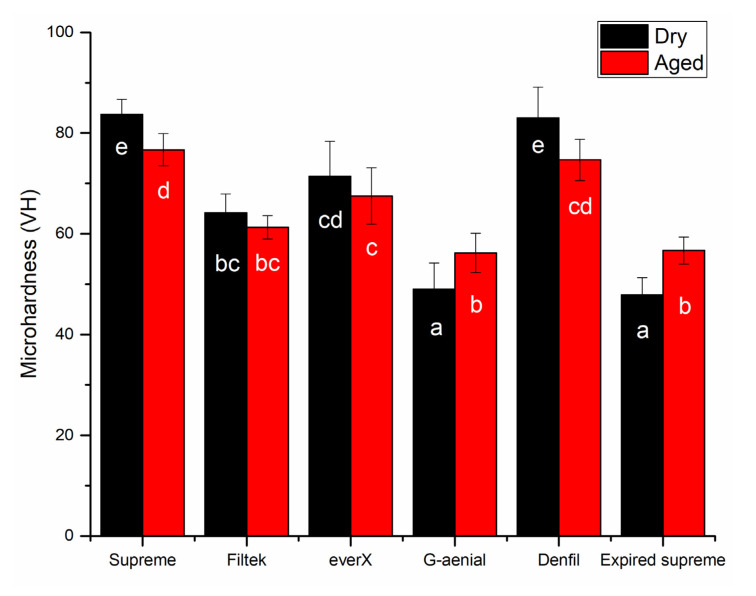
Bar graph showing means surface microhardness (VH) with standard deviations (SD) of tested resin composites. Non-statistically relevant variations (*p* > 0.05) between the materials are represented by the same letters within the bars.

**Figure 3:**
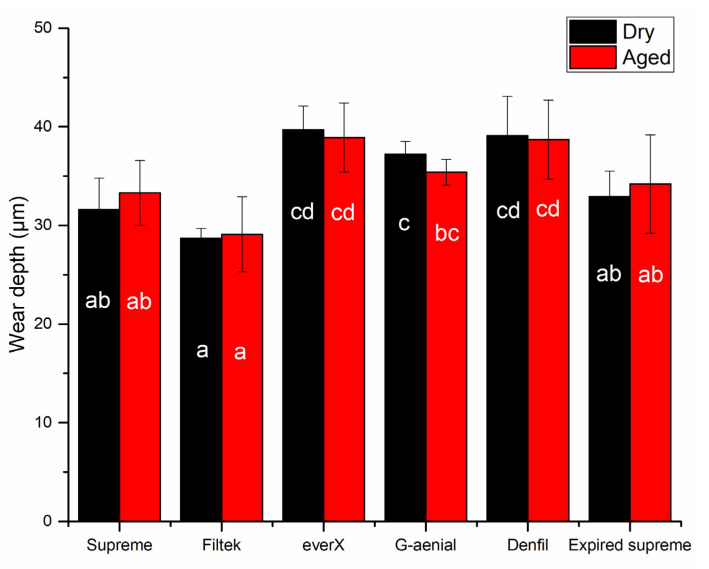
Bar graph showing means of wear depth (µm) with standard deviations (SD) of tested resin composites. Non-statistically relevant variations (*p* > 0.05) between the materials are represented by the same letters within the bars.

Denfil showed the highest DC% (59.5), Filtek Bulk Fill and everX Posterior had similar DC% values (56), while G-aenial Posterior and Supreme presented the lowest DC% values (52, 51). It was not able to test the DC% of the expired Supreme, due to detachment of the RC from the FT-IR sensor after polymerization. In general, hydrothermal accelerated aging had no significant deterioration effects on the surface microhardness (VH) and wear of investigated RCs. Meanwhile, the expiry date significantly (*p* < 0.05) decreased only the VH of supreme RC (Figure 2 and Figure 3). Supreme and Denfil in dry condition showed the highest values of surface microhardness (84, 83 VH) among the RCs tested. While G-aenial Posterior and expired Supreme presented the lowest similar values (49, 56 VH) in both conditions.

Figure 3 shows the average wear depth values for each RC after 15,000 chewing simulation cycles. Filtek Bulk Fill had the lowest average wear depth (29 µm) (*p* < 0.05), while everX Posterior and Denfil had the highest wear depth values (40, 39 µm) in both conditions. 

**Figure 4:**
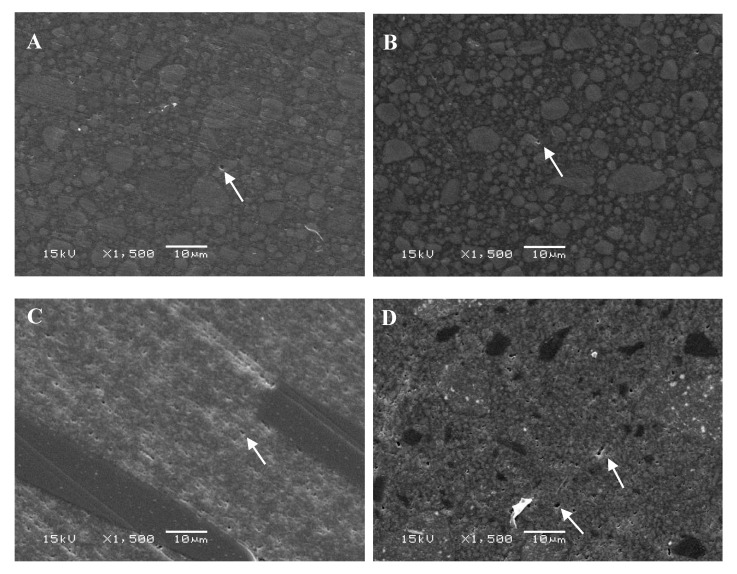

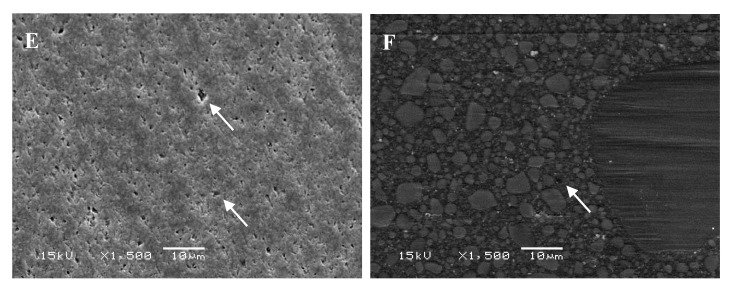
SEM photomicrographs (magnifications: 1500×) of investigated resin composites after hydrothermal accelerated aging. (**A**) Supreme; (**B**) Filtek; (**C**) everX; (**D**) G-aenial; (**E**) Denfil; (**F**) Expired supreme. Arrows indicate small pit defects.

SEM evaluation presented a conventional structure of each investigated RC after aging with different filler particulates shape and size in composite matrix (Figure 4). After ageing, Denfil revealed a significant number of small pits caused by filler particle exfoliation. These findings indicated a possible explanation for the observed differences in behavior among the RCs.

## 4. Discussion

Hydrothermal accelerated product aging is a technique that aims to assess a material’s response under normal-usage conditions over a long period of time by subjecting the product for a short period of time to moisture and stresses that are more extreme than normal environmental thermal stresses [17]. Of course, this laboratory artificial aging cannot be directly translated to the clinical situation, but it at least provides an indication of the materials’ stability. In the current research, micro-hybrid, nano-filled and short fiber-reinforced RCs were submitted to artificial hydrothermal accelerated aging by boiling of specimens in water. The length of boiling time was according to the study of Bouillaguet et al., who reported a decrease in the dental composite strength after the first 16 h of immersion specimens in boiling water [18]. Three-point bending, surface microhardness and wear test set-up were selected as reliable and relatively simple tests procedure. It has been stated earlier that under a boiling condition, standard bar-shaped composite specimens (2 × 2 × 25 mm^3^) are more prone to flaws and deformation, which can have a strong impact on the three-point bending test results [18]. Accordingly, in our study we modify the dimension of the three-point bending specimens (3 × 4 × 25 mm^3^) to overcome this limitation.

According to our results, hydrothermal accelerated aging significantly reduced the flexural properties in comparison with control condition for most of the investigated RCs. Thus, the hypothesis that accelerated aging will have no impact on the flexural properties of the tested RCs was rejected. Furthermore, this aging method could be helpful in screening and testing materials under heat and moisture stress, as well as assessing the hydrolytic stability of a dental composite.

Based on polymer engineering theory, there are three mechanisms of composite water uptake: diffusion of water molecules within the matrix, infiltration at the matrix-filler interface, and absorbing into the micro cracks produced by the action of high temperature [19]. The water uptake causes the matrix to expand, which induces stress inside the material. Furthermore, moisture increases the degradation of composite by the breaking up of molecular chains and deterioration of the interface between matrix and fillers [20]. In addition to that, high-temperature conditions such as boiling sped up the rate of moisture diffusion and resulted in the plasticization of composites [20]. 

Constantly boiling dental composite in water was previously documented to induce penetration of water into the resin matrix of composite structured dental polymers, consequently softening the polymer matrix [15,18]. Furthermore, the penetrated water was thought to induce hydrolysis of the interfacial silane bonding agent, which resulted in a weak chemical bond between the resin structure and fillers [10,21]. Thereby, the reduction in the flexural properties of the tested RCs after aging may be the results of these mechanisms [22,23]. In the structure of RCs, radiopaque fillers such as barium, strontium, and zirconium are widely used. As a result of their poor hydrolytic stability, these types of fillers, especially barium glass, can cause a decrease in flexural property [22,23]. On the contrary, the non-significant change in flexural strength of G-aenial Posterior after accelerated aging could be explained by its high fillers content, in particular the large sized pre-polymerized fillers (Figure 4D). Another possible cause is that during immersion in 100 °C water, the degree of monomer conversion (DC%) increased, i.e., the post curing effect [8]. Among the tested RCs, there were differences in the tested properties. Because of variations in polymerization initiation processes, filler structure, and resin matrix, the values varied greatly.

Among the investigated RCs, Filtek Bulk Fill and everX Posterior showed the highest values of flexural strength and modulus, which appears to be the outcome of good light transmission and curing through these composites [24]. everX Posterior reinforced by millimeter scale short fibers (Ø17 μm) had the desired fiber aspect ratio and reinforcing capability [9]. Therefore, the finding of improved flexural performance was expected. On the other hand, Denfil with a higher filler loading (83 wt%) showed lower flexural performance than other RCs, which have a lower percentage of filler loading (Table 1). In other terms, this research verified the lack of a clear link between percentage of filler loading and flexural performance.

In addition to mechanical properties, surface microhardness and wear are important factors that have to be considered in the selection of restorative materials. Interestingly, accelerated aging in this study showed no significant impacts on the surface microhardness and wear of investigated RCs. Therefore, the hypothesis that accelerated aging would have no effect on the surface properties of the investigated RCs was accepted.

It seems that filler particles are more critical for surface microhardness and wear resistance in composite materials than the matrix system. According to McCabe and Wassell, microhardness of composite materials enhanced with increasing filler content [25]. Our results are in line with this statement as Denfil with high inorganic filler content (83 wt%) had the highest surface microhardness values (Figure 2). According to Condon and Ferracane, the impact of filler volume on wear resistance is linear, with high filler volumes lowering the wear rates owing to the reduced amount of resin unreinforced by particulate fillers [26]. However, in the current research, there was no link found between filler content and two-body wear as Denfil showed the highest wear depth values in both conditions, which is in line with some reported data in literature [27,28,29]. In fact, the morphology, type, chemistry, and filler size have also been reported to have an impact on the composite microhardness and wear efficiency [29,30,31]. In line with previous study, short fiber-reinforced RC showed lower wear resistance than other RCs (Figure 3) [32]. However, it should be noted that everX Posterior is instructed to be covered from all surfaces with a layer of conventional RC.

Our findings are consistent with previous theoretical and laboratory research, which found that restorative composite materials with small filler particles have increased surface microhardness. Supreme had partially nano-sized silica fillers (Ø 20 nm) and this could justify the higher surface microhardness values. On the other side, G-aenial Posterior presented lower surface microhardness and DC% values than all the other tested RCs. The finding is in accordance with earlier research, which showed that microhybrid G-aenial composite has lower DC% and surface microhardness values than other conventional and bulk-fill RCs [24].

Although using expired dental resin composites is not recommended, some dentists may use these materials by accident or on purpose due to the high cost of resin composites. Therefore, evaluating the mechanical properties and wear of these resin composites is important in relation to the materials’ clinical use. Surprisingly, the time aging (>3 year expiry) does not affect the flexural properties and wear of the tested RC (Supreme XTE). This is in accordance with few reports in literature, which showed that some composites retained baseline flexure strength and modulus for up to 30 months after their expiration date [33,34]. On the other hand, surface microhardness of expired supreme showed dramatic reduction along VH and it dropped by 43% from unexpired material (Figure 2). This possibly explained the presence of thick oxygen inhibition layer of unpolymrized monomer on the surface of expired composite [35]. Unfortunately, we were not able to measure the DC% of the expired RC because of the specimen’s detachment after polymerization, which could be a result of high volumetric shrinkage and induced shrinkage stress.

It is important to highlight that in this research, only some mechanical and surface performance of expired composite was investigated, other clinically important parameters such as polymerization shrinkage stress, water sorption/solubility and cytotoxicity of expired material were not evaluated.

## 5. Conclusions

Within the limitations of this study, it can be concluded that:

1. Hydrothermal accelerated aging decreased the flexural strength compared to the control condition for the tested RCs except for G-aenial Posterior, which showed no differences.
2. Hydrothermal accelerated aging has no influence on surface microhardness and the wear of tested RCs.
3. Passing the expiration date for 40 months did not affect the flexural strength and wear of tested RC. Conversely, this time aging seemed to have a negative effect on the surface microhardness.

## Figures and Tables

**Table 1:** The packable resin composites tested in this research.

Brand	Manufacturer	Type	Composition
Supreme XTE	3M/ESPE, St. Paul, MN, USA	Nano-filled	Bis-GMA, UDMA, TEGDMA, Bis-EMA, 78.5 wt% Zirconia/silica cluster and silica fillers (av. Ø 20 nm)
Filtek Bulk Fill	3M/ESPE, St. Paul, MN, USA	Nano-filled	AUDMA, UDMA, DDDMA, 76.5 wt% Zirconia/silica and ytterbium trifluoride fillers in nanometer scale (av. Ø 20 nm)
everX Posterior	GC Corp, Tokyo, Japan	Short fiber-reinforced	Bis-GMA, PMMA, TEGDMA, millimetre scale glass fiber filler, Barium glass 76 wt%, 57 vol%
G-aenial Posterior	GC Corp, Tokyo, Japan	Micro-hybrid	UDMA, dimethacrylate co-monomers, Prepolymerized silica and strontium fluoride containing fillers 80 wt%
Denfil	Vericom Corp., Korea	Micro-hybrid	Bis-GMA, TEGDMA, 80 wt% Barium aluminosilicate, Fumed silica

Bis-GMA, bisphenol-A-glycidyl dimethacrylate; UDMA, urethane dimethacrylate; TEGDMA, triethylene glycol dimethacrylate; AUDMA, Aromatic urethane dimethacrylate; DDDMA, 12-dodecanediol dimethacrylate; Bis-EMA, Ethoxylated bisphenol-A-dimethacrylate; PMMA, polymethylmethacrylate; wt%, weight percentage; vol%, volume percentage.

## Data Availability

Data sharing is not applicable to this article.
